# 

**Published:** 1843-10-28

**Authors:** 


### CONTENTS.

A Course of Clinical Lectures, delivered at the Hôtel Dieu, Paris, for the Session 1842–'43. By A. F. Chomel, M.D. Lecture 14, - - - - - - 241
Case of Spinal Irritation giving rise to symptoms simulating Catalepsy. By George L. Upshur, M. D., - - 243
*Bibliographical Notice.*
A Practical Treatise on the Diseases of the Testis and of the Spermatic Cord and Scrotum, with Illustrations. By T. B. Curling, Lecturer on Surgery, &c. Edited by P. B. Goddard, M.D. 243
EDITORIAL,
Statue of Bichat, at Bourg, - - 244
Death of Dr. Jacobson, of Copenhagen, 244
Death of Dr. Harlan, - - - - 245
*Retrospect of the Medical Sciences.*
Adulteration of Common Salt at Paris, - 245
Scott's New Knife for the Operation of Cataract, - - - - - - 245
Narrow Escape of a Druggist, - - 245
On the [alleged] Cure of Pulmonary Consumption with Naphtha. By J. Hastings, M. D., - - - - - 246
Varus Mentagra and Gutta Rosea, the Sycosis Menti and Acne of Willan, Treated with Sulphate of Iron Externally. By W. Dauverger, - - 247
Convenient Mode of Administering Oil of Turpentine. By M. Bouchardat, - 247
Frequency of Criminal Abortion, - 247
Drs. Haplin and Johns on Puerperal Convulsions, - - - - - - 248
Delirium Tremens. By H. Johnson, M.D. 248
Mortality after Amputation, - - 249
Observatipns on the Muscle of the Lacrymal Sac, or Tensor Tarsi, - 249
A Caution Respecting Nitric Acid, - 249
Dr. Christison on Fluid Extract of Senna, 250
Dr. Ure on a New Solvent for Stone in the Bladder, - - - - 250
Dr. Simon on Urine in Typhus, - - 251
Urine in Scarlatina. By Dr. Simon, - 252

